# Impaired bone health after pediatric acute lymphoblastic leukemia treatment despite normal DXA: insights from high-resolution peripheral quantitative computed tomography

**DOI:** 10.3389/fendo.2026.1850461

**Published:** 2026-09-07

**Authors:** Nina Dominique Stahel, Nicole Bodmer, Aziz Chaouch, Etienne Sochett, Flavia M. Wehrle, Marianne Rohrbach, Camilla Saladin, HansJörg Häuselmann, Daniel Konrad, Nina Lenherr-Taube

**Affiliations:** 1 Division of Pediatric Endocrinology, University Children’s Hospital Zurich and Children’s Research Center, University Zurich, Zurich, Switzerland; 2 Division of Oncology, University Children’s Hospital, University Zurich, Zurich, Switzerland; 3 Department of Epidemiology and Health Systems, Quantitative Research, Center for Primary Care and Public Health (Unisanté), University of Lausanne, Lausanne, Switzerland; 4 Department of Pediatrics, Division of Endocrinology, The Hospital for Sick Children, University of Toronto, Toronto, ON, Canada; 5 Child Development Center, University Children’s Hospital Zurich and Children’s Research Center, University Zurich, Zurich, Switzerland; 6 Division of Metabolism, Connective Tissue Unit, University Children’s Hospital and Children’s Research Center, University Zurich, Zurich, Switzerland; 7 Division of Radiology, University Children’s Hospital and Children’s Research Center, University Zurich, Zurich, Switzerland; 8 Center for Rheumatology and Bone Disease, Zurich, Switzerland

**Keywords:** ALL survivor, dual-energy x-ray absorptiometry, HR-pQCT, osteoporosis, vertebral fractures

## Abstract

**Introduction:**

Acute lymphoblastic leukemia (ALL) is the most common cancer in childhood. With improved survival rates, more attention has turned to long-term outcomes such as bone health. High-resolution peripheral quantitative computed tomography (HR-pQCT) allows a three-dimensional assessment of cortical and trabecular bone and may have advantages over standard dual-energy X-ray absorptiometry (DXA). The aim of this study was to evaluate whether HR-pQCT can detect early bone health impairment in children following ALL treatment.

**Methods:**

In this cross-sectional study, HR-pQCT findings in children within 5 years after completion of ALL therapy were compared with healthy controls. Additional assessments for ALL patients included lumbar spine bone mineral density (LSBMD) assessed by DXA and vertebral fracture (VF) assessment using EOS.

**Results:**

HR-pQCT data from 30 ALL patients (14 male patients; median age 8.8 years) and 64 healthy controls (30 male controls; median age 9.4 years) were analyzed. Five ALL patients (17%) showed evidence of one or more VFs. LSBMD z-scores were within the normal range for all patients (mean 0.09 SDS). Compared with healthy controls, children after ALL treatment showed significantly reduced cortical area (p = 0.013), cortical BMD (p = 0.001), and cortical thickness (p = 0.006) at the tibia, and reduced cortical area (p = 0.046) and cortical BMD (p = 0.008) at the radius on HR-pQCT.

**Conclusion:**

Despite normal LSBMD DXA z-scores, a substantial proportion of pediatric ALL patients had vertebral fractures and lower values for several cortical parameters at both the radius and tibia on HR-pQCT. These findings suggest that HR-pQCT may serve as a valuable adjunctive tool to detect bone health impairment in children after ALL treatment.

## 1 Introduction

Acute lymphoblastic leukemia (ALL) is the most common cancer in childhood and is reported to affect approximately 1 in 2000 children between 0 and 15 years (1). Over the past decades, cure rates have approached 90%, and with improved survival, attention has turned toward long-term outcomes (2).

Bone mass development is a critical aspect of childhood as peak bone mass is typically achieved in the late adolescent years (3). It is a key determinant of lifelong bone strength, with higher peak values associated with a reduced risk for osteoporosis and fractures later in life (4, 5). The effects of ALL, its treatment, and complications make the growing skeleton especially vulnerable through suppression of bone formation and accelerated bone resorption (6, 7). More specifically, steroid exposure, osteotoxic chemotherapy, poor nutrition, low vitamin D levels, low physical activity during ALL treatment, and the direct impact of leukemia on the skeleton may result in osteoporosis, osteonecrosis, and fractures in this at-risk population (8, 9). However, to date, there are only a few studies investigating bone health in children after ALL treatment (10–14). A Canadian multicenter study documented a cumulative incidence of vertebral fractures (VF) of 26% over the 4 years after diagnosis of ALL (12). Similar results were shown in three other studies with a cumulative fracture incidence of 17.8% from diagnosis until 1 year after the end of therapy (10), a 5-year incidence of 32.7% for skeletal complications (13), and a fracture rate that was 6 times higher in pediatric ALL patients than in healthy controls (14).

Therefore, the International Guideline Harmonization Group for Late Effects of Childhood Cancer has identified bone health as a priority for future guideline development. Current aftercare protocols regarding bone health screening are inconsistent. Some protocols suggest evaluating bone health with dual-energy X-ray absorptiometry (DXA) every 2–10 years in addition to bone health-related biochemical testing to varying degrees. DXA is the standard diagnostic tool to assess bone mineral density (BMD) in children at risk for impaired bone health (15). A major limitation of this method is the two-dimensional representation of areal BMD, which is dependent on the patient’s height and pubertal stage, both of which can be affected by a chronic disease such as ALL. Further, DXA cannot differentiate between trabecular and cortical bone and may therefore fail to identify subthreshold changes in these compartments. For this reason, DXA may be unable to accurately identify all patients at risk for bone health complications (16, 17).

High-resolution peripheral quantitative computed tomography (HR-pQCT) is an established diagnostic research tool to assess bone health and provides a detailed three-dimensional volumetric assessment of both cortical and trabecular bone rather than the areal BMD assessment provided by DXA (18). Whereas DXA can only assess bone mass, HR-pQCT provides a more comprehensive assessment of bone quality and strength: in addition to volumetric BMD, HR-pQCT allows for measurement of parameters corresponding to those previously attainable only by invasive bone biopsy, such as trabecular bone volume fraction, trabecular number, trabecular thickness, and trabecular separation, and cortical parameters (19).

In the adult general population, HR-pQCT parameters such as cortical BMD, cortical thickness, and failure load have been identified as independent predictors of incident fracture risk (20–22). Similar studies have not yet been performed in children, and published normative data for this age group are still limited (23, 24).

Consequently, the aim of this study was to compare bone microarchitecture parameters measured using HR-pQCT between a cohort of children after ALL treatment and age- and sex-comparable healthy controls and to explore potential differences in HR-pQCT findings between the two groups.

## 2 Methods

### 2.1 Study design and study population

This is an observational single-center cross-sectional study including 102 children recruited between October 2021 and October 2024 at the University Children’s Hospital Zurich. Inclusion criteria for this study were age 5–18 years at the time of enrollment, written informed consent from the participant and/or parent/legal guardian, and, for patients, a diagnosis of ALL and completion of ALL therapy within the previous 5 years. Exclusion criteria were the presence of any other significant medical or surgical disorder that could influence bone health, such as long-term use of steroids before ALL diagnosis, impaired mobility, or thyroid disease. A total of 34 pediatric ALL patients fulfilled the inclusion criteria and consented to participate. In addition, 68 healthy controls with a similar age and sex distribution were recruited. One pediatric ALL patient withdrew consent after the screening visit, and one was excluded due to the occurrence of a secondary tumor. After HR-pQCT quality control, two ALL patients and two healthy controls were excluded due to motion artifacts. As previous literature has demonstrated ethnic differences in HR-pQCT (25), two healthy controls with mixed ethnicity were excluded from further analysis to match the Caucasian ethnicity of the remaining pediatric ALL patients. Thus, 30 ALL survivors and 64 healthy controls were included in the final analysis.

The ethics committee of the Canton of Zurich, Switzerland, approved this study (BASEC No. 2021-01033), and the study was performed in accordance with the WMA Declaration of Helsinki. Informed consent from participants and their legal guardians was obtained.

### 2.2 Clinical characteristics and laboratory measures

Demographic data including age, sex, and ethnicity were obtained. Height was measured using a wall-mounted stadiometer, and weight was assessed using an electronic scale. Body mass index (BMI) was calculated based on these measures. A physical pubertal exam (Tanner staging) was performed by an experienced pediatrician in all pediatric ALL patients within 6 months of study entry, and bone age was assessed from a hand X-ray using BoneXpert software version 3.2.3 (algorithm v1.7.3) for all participants (26). All participants completed a food and supplement record to assess dietary calcium and vitamin D intake, which was reported as a percentage of the dietary reference intake (DRI) for age (27, 28). Moderate-to-vigorous physical activity (MVPA) for each participant was calculated using a validated physical activity questionnaire (29). The presence or absence of back pain was recorded, and fracture history was obtained. For children and adolescents after ALL therapy, serum calcium, phosphate, magnesium, creatinine, alkaline phosphatase, 25-hydroxy vitamin D, and intact parathyroid hormone (PTH), together with the urinary calcium-to-creatinine ratio, were measured in the Department of Laboratory Medicine at the University Children’s Hospital Zurich within 6 months of study entry. Determination of vitamin D deficiency was based on the Global Consensus Recommendations on Prevention and Management of Nutritional Rickets (27).

### 2.3 HR-pQCT imaging and analysis

HR-pQCT measurements were performed by HJH at the Center for Rheumatology and Bone Disease (ZRK), Zurich, Switzerland, using an XtremeCTI scanner (Scanco Medical AG, Brüttisellen, Switzerland) following the recently published guidelines (30). The non-dominant radius and the right tibia were imaged in all participants. The participant’s forearm was placed in a carbon fiber cast that was specifically manufactured by Scanco Medical AG for children. For the ankle, the smallest adult carbon fiber cast was used.

We used the fixed offset method of 2 mm from the reference line in cases of an open or visibly fused growth plate (GP). The operator placed this reference line at the proximal medial edge of the GP of the distal radius and tibia as previously described (24). The scan region began 2 mm proximal to the reference line for the radius and tibia (31–33). The scan region then extended proximally from this point by 9.02 mm (110 slices) for first-generation scanners (31–33). All scans were graded with regard to subject motion using the five-level motion grading scale (best score, 1; worst score, 5) as described elsewhere (30). If motion artifacts with a score of three or more were observed, the scans were excluded as recommended.

Standard morphologic analysis was used to measure volumetric average BMD (D100; mg HA/cm³) and trabecular BMD (Dtrab; mg HA/cm³), meta trabecular BMD (outer layer of trabecular BMD) (Dmeta; mg HA/cm³), inner trabecular BMD (inner layer of trabecular BMD) (Dinn; mg HA/cm³), ratio meta to inner trabecular BMD (Meta/Inn), trabecular number (Tb.N; mm^-1^), trabecular thickness (Tb.Th; mm), trabecular separation (Tb.Sp; mm), trabecular bone volume fraction (BV/TV), total area (Tt.Ar; mm²), trabecular area (Tb.Ar; mm²), cortical BMD (Dcomp; mg HA/cm³), cortical thickness (Ct.Th; mm), cortical area (Ct.Ar; mm²), cortical perimeter (Ct.Pm; mm), cortical pore diameter (Ct.Po.Dm; mm) and cortical porosity (Co.Po; %) (33). Trabecular thickness (Tb.Th; mm), trabecular separation (Tb.Sp; mm), and trabecular separation standard deviation (Tb/N.SD; mm) were calculated using an automated segmentation method (34).

#### 2.3.1 Finite-element analysis

Linear finite-element analysis models were created from the HR-pQCT images using the software provided by the manufacturer (uFE analysis solver v.1.15, Scanco Medical AG) to evaluate bone strength (failure load; newtons) as described elsewhere (33).

### 2.4 Dual-energy X-ray absorptiometry

Lumbar spine (L1 to L4) areal BMD (LSBMD) was measured for all pediatric ALL patients in the anteroposterior projection using dual-energy X-ray absorptiometry (DXA; GE Lunar systems, GE Healthcare, Madison, WI, USA). Areal BMD values were converted to z-scores using equipment-specific age- and sex-adjusted reference data provided by the manufacturer (Encore 2011 software, version 13.60.033) (35, 36). Further, height-adjusted LSBMD z-scores were generated using the Hologic-specific reference values after transforming BMD results from Lunar to Hologic as suggested by the manufacturer (37).

### 2.5 Lateral X-ray spine (EOS) and vertebral fracture assessment

Low-dose biplanar radiographic imaging was performed using the EOS system (EOS imaging, Paris, France; EOSedge since 14 November 2024; previously EOS-System). Lateral spine imaging was obtained for all pediatric ALL patients, and VF assessment was performed by two independent experienced pediatric radiologists, including CS, using the Genant semiquantitative method (38).

### 2.6 Statistical analysis

Demographic information was presented as counts and percentages for categorical variables, while continuous variables were summarized using mean and standard deviation (SD) or median and interquartile range (IQR), as appropriate. Group differences in continuous variables were tested using the Welch’s t-test (normal distribution) or the Mann–Whitney U test (non-normal distribution). Chi-square tests or Fisher’s exact tests were used to test group differences in categorical variables.

Unadjusted comparisons of HR-pQCT parameters between the two groups were also tested using Welch’s t-test or the Mann–Whitney U test, separately for the radius and tibia. In addition to uncorrected p-values, a correction for multiple testing using the Holm method (39) was applied to report adjusted p-values.

In-sample reference curves (i.e., norms) for HR-pQCT parameters were constructed using data from the 64 healthy controls. More specifically, radius and tibia measurements from healthy subjects were analyzed jointly in linear mixed models (40) as a function of age and sex, including site (radius/tibia) as a fixed effect and a random intercept for each subject. A suitable transformation for each HR-pQCT parameter was selected using the Box–Cox transformation (41). Model selection was performed by minimizing the Bayesian Information Criterion (BIC; 42), which applies a stronger penalty to complex predictive models compared with the Akaike Information Criterion (AIC; 43). In particular, age was modeled using penalized splines (44) in order to accommodate possibly non-linear patterns when this resulted in an improved fit (i.e., lower BIC). Male and female participants were modeled jointly, introducing interactions between age and sex only when supported by the data (i.e., resulting in a lower BIC compared with a model without interaction). The norms generated from these models for all HR-pQCT parameters were then used to compute z-scores for both healthy controls and ALL survivors and to calculate the mean difference between the groups.

As only 64 healthy controls were used to generate the reference curves, a non-parametric bootstrap (45) was used to account for the uncertainty in the estimation of z-scores when quantifying the difference between the two groups. The bootstrap first resampled subjects within control and ALL groups, refitted the norms to the resampled controls, and then re-estimated the difference between the average z-scores in the two groups. The process was repeated 2,000 times in order to build 95% confidence intervals for the difference in z-scores, with and without stratification by sex or age groups. Z-scores for HR-pQCT parameters of pediatric ALL patients with and without VF and for prepubertal and pubertal ALL patients were shown visually using stripcharts. Due to the small sample size in these subgroups, no statistical test was performed and only a visual inspection was carried out.

All statistical analyses were carried out in R version 4.2.2 (46) with a level of statistical significance set at 5%.

The demographic and clinical characteristics of 30 pediatric ALL patients (median age 8.8; min. 6, max. 17 years, 16 female patients, 14 male patients) and 64 healthy controls (median age 9.4; min. 5, max. 17 years, 34 female controls, 30 male controls) are summarized in **Table 1**. Both cohorts were Caucasian. In the ALL group, 22 patients were prepubertal (Tanner stage 1), and 8 patients had entered puberty at the time of the study visit (Tanner stage 2–4). Bone age at study entry was comparable between both groups, with a mean difference between age and bone age of -0.11 years for ALL patients and 0.29 years for healthy controls (p = 0.063). Mean BMI z-score in pediatric ALL patients was higher than that in healthy controls (0.77 versus 0.14, p = 0.003). No relevant differences were shown in back pain prevalence, dietary calcium intake, or MVPA. In contrast, although the difference did not reach statistical significance, the proportion of participants taking vitamin D supplements was more than twice as high in the ALL group as in healthy controls (30% versus 12.5%, p = 0.077).

**Table 1:** Cohort characteristics of ALL survivors and healthy controls.

Variables	Controls (N=64)	ALL (N=30)	p
Demographics			
Sex = Male (%)	30 (46.9)	14 (46.7)	1.000
Chronological age (years) [median (IQR)]	9.38 [7.12, 12.42]	8.78 [7.14, 11.30]	0.958
Bone age (years) [median (IQR)]	9.16 [7.00, 12.70]	8.68 [7.49, 11.89]	0.736
Difference between chronological and bone age (years) [mean (SD)]	0.29 (0.87)	-0.11 (1.15)	0.063
Weight z-score [mean (SD)]	0.34 (0.79)	0.64 (1.20)	0.144
Height z-score [mean (SD)]	0.55 (0.94)	0.24 (1.01)	0.155
BMI z-score [mean (SD)]	0.14 (0.85)	0.77 (1.13)	0.003
Prepubertal (Tanner Stage 1)(%)	NA	22 (73.4)	NA
Bone health questionnaire			
Back pain in last 12 months = At least 1 time (%)	10 (15.6)	6 (20.0)	0.817
History of peripheral fractures = 1 or more peripheral fracture(s) (%)	10 (15.6)	2 ( 6.7)	0.326
Taking vitamin D supplements = Yes (%)	8 (12.5)	9 (30.0)	0.077
DRI Calcium = <100% (%)	62 (96.9)	29 (96.7)	1.000
MVPA (min/day) = >=60 min (%)	39 (60.9)	21 (70.0)	0.534

Categorical variables were summarized using counts and percentages, with groups compared using chi-square or Fisher exact tests.

Continuous variables were summarized using either mean and standard deviation (SD), or using the median and interquartile range (IQR), with groups compared using student's t-tests or Mann-Whitey U tests.

## 3 Results

### 3.1 Cohort characteristics

ALL disease characteristics and clinical bone health workup for pediatric ALL patients are shown in **Table 2**. The majority of patients were diagnosed with common B-ALL (4 high-risk and 25 non-high-risk) and were included in this study a mean of 2.6 years (min. 0; max. 5) after the end of therapy. Cumulative glucocorticoid exposure is provided for each disease group. All patients were treated according to the AIEOP-BFM ALL 2017 protocol (International Collaborative Treatment Protocol for Children and Adolescents with Acute Lymphoblastic Leukemia). The cumulative doxorubicin dose was 150–180 mg/m² for non-high-risk patients and 240–255 mg/m² for high-risk patients. The cumulative dose of methotrexate was 20 g/m² for the non-high-risk group and 10 g/m² for the high-risk group. Five of the 30 patients had evidence of at least one VF on EOS imaging, confirming osteoporosis. LSBMD z-scores from DXA were within the normal range for all patients (mean z-score -0.09, SD = 0.85), including when adjusted for height (mean height-adjusted z-score 0.27, SD = 0.88). Biochemical bone health measures were generally within the normal range for age. Five pediatric ALL patients had vitamin D deficiency (26.6–46.7 nmol/L, reference >50), and none of them showed elevated PTH.

**Table 2:** ALL disease characteristics and general bone health assessment.

Variables	ALL (N=30)	Reference range
Characteristics of Leukemia		
ALL risk category = High risk (%)	4 (13.3)	
ALL diagnosis = B-ALL (%)	29 (96.7)	
Cumulative glucocorticoid doses (mg/m2 prednisone equivalent)		
- p B-ALL non-high risk (n=25)	3413	
- T-ALL non-high risk (n=1)	3483	
- B-ALL high risk (n=4)	5725	
Years after therapy end [mean (SD)]	2.60 (1.71)	
Vertebral fracture = yes (%)	5 (16.7)	0
LSBMD z-score [mean (SD)]	-0.09 (0.85)	-2-2
LSBMD z-score from DXA corrected for height [mean (SD)]	0.27 (0.88)	
Laboratory measures		
Calcium (mmol/L) [mean (SD)]	2.36 (0.07)	2.10-2.70
Phosphate (mmol/L) [mean (SD)]	1.36 (0.21)	1.0-1.7
Magnesium (mmol/L) [mean (SD)]	0.85 (0.05)	0.74-1.03
PTH (mU/L) [mean (SD)]	43.57 (13.21)	15-65
Creatinine (umol/L) [median (IQR)]	36.00 [32.00, 43.75]	<60
ALP (U/L) [mean (SD)]	226.73 (77.36)	265
25-OHD (nmol/L) [mean (SD)]	77.59 (29.04)	>50
Urine Ca/Cr (mmol/mmol) [median (IQR)]	0.15 [0.00, 0.34]	0.04-0.7

Categorical variables were summarized using counts and percentages, with groups compared using chi-square or Fisher exact tests.

Continuous variables were summarized using either mean and standard deviation (SD), or using the median and interquartile range (IQR), with groups compared using student's t-tests or Mann-Whitey U tests.

### 3.2 HR-pQCT parameters for the tibia and radius

HR-pQCT parameters for ALL survivors and healthy controls are provided in **Table 3a** for the tibia and in **Table 3b** for the radius. Pediatric ALL patients showed impairment in several parameters, mainly cortical parameters, at the tibia and in two parameters at the radius compared with age- and sex-comparable healthy controls. However, after correction for multiple testing, only the bone density parameters cortical BMD and the ratio of meta to inner trabecular BMD at the tibia showed statistically significant reductions in ALL patients compared with the healthy controls (p = 0.019).

**Table 3a:** HR-pQCT results of the tibia.

Outcome	Control (N=64)	ALL (N=30)	p	p*
Bone geometry				
Tt.Ar (mm²) [median (IQR)]	661.35 [499.35, 850.52]	638.55 [528.22, 852.70]	0.739	1
Ct.Ar (mm²) [median (IQR)]	27.85 [17.68, 39.50]	20.40 [10.68, 29.02]	0.013	0.208
Tb.Ar (mm²) [median (IQR)]	609.60 [458.05, 782.05]	586.35 [460.75, 788.52]	0.715	1
Bone density				
D100 (mg HA/cm³) [median (IQR)]	235.31 (32.71)	231.21 (33.38)	0.579	1
Dcomp (mg HA/cm³) [median (IQR)]	629.75 [589.65, 671.62]	578.65 [528.70, 622.05]	0.001	0.019
Dtrab (mg HA/cm³) [mean (SD)]	199.97 (28.56)	203.87 (31.53)	0.566	1
Dmeta (mg HA/cm³) [mean (SD)]	249.07 (33.02)	238.26 (39.62)	0.200	1
Dinn (mg HA/cm³) [mean (SD)]	166.58 (28.44)	180.53 (33.48)	0.054	0.756
Meta/Inn [median (IQR)]	1.49 [1.39, 1.61]	1.37 [1.23, 1.50]	0.001	0.019
Bone micro architecture				
BV/TV [mean (SD)]	0.17 (0.02)	0.17 (0.03)	0.563	1
Ct.Th (mm) [median (IQR)]	0.55 [0.46, 0.62]	0.47 [0.41, 0.55]	0.006	0.102
Ct.Pm (mm) [median (IQR)]	102.10 [88.78, 116.02]	100.45 [90.02, 116.70]	0.862	1
Ct.Po (%) [median (IQR)]	3.30 [2.40, 4.03]	3.10 [2.50, 3.80]	0.545	1
Ct.Po.Dm (mm) [median (IQR)]	0.14 [0.14, 0.15]	0.14 [0.14, 0.15]	0.017	0.255
Tb.N (1/mm) [mean (SD)]	2.32 (0.18)	2.31 (0.20)	0.840	1
Tb.Th (mm) [median (IQR)]	0.07 [0.07, 0.08]	0.07 [0.07, 0.08]	0.394	1
Tb.Sp (mm) [median (IQR)]	0.36 [0.34, 0.39]	0.36 [0.33, 0.40]	0.984	1
Tb/N.SD (mm) [median (IQR)]	0.14 [0.12, 0.16]	0.15 [0.13, 0.17]	0.164	1
Estimated bone strength				
Failure load (N) [median (IQR)]	5797.35 [4806.43, 8331.35]	5382.66 [4004.04, 6672.00]	0.136	1

Tt.Ar, total area; Ct.Ar, cortical area; Tb.Ar, trabecular area; D100, volumetric average BMD; Dcomp, cortical BMD; Dtrab, trabecular BMD; Dmeta, meta trabecular BMD; Dinn, inner trabecular BMD; Meta/Inn, ratio meta to inner trabecular BMD; BV/TV, trabecular bone volume fraction; Ct.Th, cortical thickness; Ct.Pm, cortical perimeter; Ct.Po, cortical porosity; Ct.Po.Dm, cortical pore diameter; Tb.N, trabecular number; Tb.Th, trabecular thickness; Tb.Sp, trabecular separation; Tb/N.SD, trabecular separation standard deviation.

Normally distributed outcomes are summarized using mean and standard deviation (SD) while non-normal outcomes are summarized using median and inter-quartile range (IQR). Between-group differences were tested using a Welch t-test for normally distributed outcomes, and a Mann-Whitney U test for non-normal outcomes. Columns with p* refer to p-values adjusted for multiple testing using the Holm method (Holm, 1979).

**Table 3b:** HR-pQCT results of the radius.

Outcome	Control (N=64)	ALL (N=30)	p	p*
Bone geometry				
Tt.Ar (mm²) [median (IQR)]	184.25 [140.55, 242.12]	191.20 [149.35, 244.65]	0.607	1
Ct.Ar (mm²) [median (IQR)]	13.45 [8.55, 18.50]	8.45 [6.25, 13.88]	0.046	0.828
Tb.Ar (mm²) [median (IQR)]	156.00 [117.90, 211.20]	163.95 [126.85, 216.07]	0.519	1
Bone density				
D100 (mg HA/cm³) [mean (SD)]	252.81 (39.02)	241.22 (38.46)	0.180	1
Dcomp (mg HA/cm³) [median (IQR)]	622.10 [565.55, 685.75]	569.95 [533.07, 601.38]	0.008	0.152
Dtrab (mg HA/cm³) [mean (SD)]	182.33 (32.64)	185.10 (36.39)	0.724	1
Dmeta (mg HA/cm³) [mean (SD)]	251.87 (33.71)	255.07 (38.09)	0.695	1
Dinn (mg HA/cm³) [mean (SD)]	134.06 (34.30)	136.60 (38.49)	0.759	1
Meta/Inn [median (IQR)]	1.96 [1.72, 2.10]	1.88 [1.78, 2.15]	0.770	1
Bone micro architecture				
BV/TV [mean (SD)]	0.15 (0.03)	0.15 (0.03)	0.740	1
Ct.Th (mm) [median (IQR)]	0.58 [0.49, 0.68]	0.52 [0.44, 0.60]	0.070	1
Ct.Pm (mm) [median (IQR)]	54.45 [47.48, 64.82]	56.95 [49.33, 63.92]	0.330	1
Ct.Po (%) [median (IQR)]	2.10 [1.80, 3.12]	2.75 [2.05, 3.80]	0.058	0.986
Ct.Po.Dm (mm) [median (IQR)]	0.14 [0.14, 0.14]	0.14 [0.14, 0.14]	0.592	1
Tb.N (1/mm) [mean (SD)]	2.11 (0.22)	2.05 (0.27)	0.309	1
Tb.Th (mm) [median (IQR)]	0.07 [0.07, 0.07]	0.07 [0.07, 0.08]	0.132	1
Tb.Sp (mm) [median (IQR)]	0.40 [0.37, 0.44]	0.40 [0.38, 0.44]	0.682	1
Tb/N.SD (mm) [median (IQR)]	0.16 [0.14, 0.18]	0.16 [0.15, 0.19]	0.279	1
Estimated bone strength				
Failure load (N) [median (IQR)]	1970.35 [1570.76, 2442.99]	1671.85 [1265.04, 2172.33]	0.098	1

Tt.Ar, total area; Ct.Ar, cortical area; Tb.Ar, trabecular area; D100, volumetric average BMD; Dcomp, cortical BMD; Dtrab, trabecular BMD; Dmeta, meta trabecular BMD; Dinn, inner trabecular BMD; Meta/Inn, ratio meta to inner trabecular BMD; BV/TV, trabecular bone volume fraction; Ct.Th, cortical thickness; Ct.Pm, cortical perimeter; Ct.Po, cortical porosity; Ct.Po.Dm, cortical pore diameter; Tb.N, trabecular number; Tb.Th, trabecular thickness; Tb.Sp, trabecular separation; Tb/N.SD, trabecular separation standard deviation.

Normally distributed outcomes are summarized using mean and standard deviation (SD) while non-normal outcomes are summarized using median and inter-quartile range (IQR). Between-group differences were tested using a Welch t-test for normally distributed outcomes, and a Mann-Whitney U test for non-normal outcomes. Columns with p* refer to p-values adjusted for multiple testing using the Holm method (Holm, 1979).

### 3.3 HR-pQCT z-scores overall, stratified by age, sex, pubertal stage, and VF

**Figure 1:**
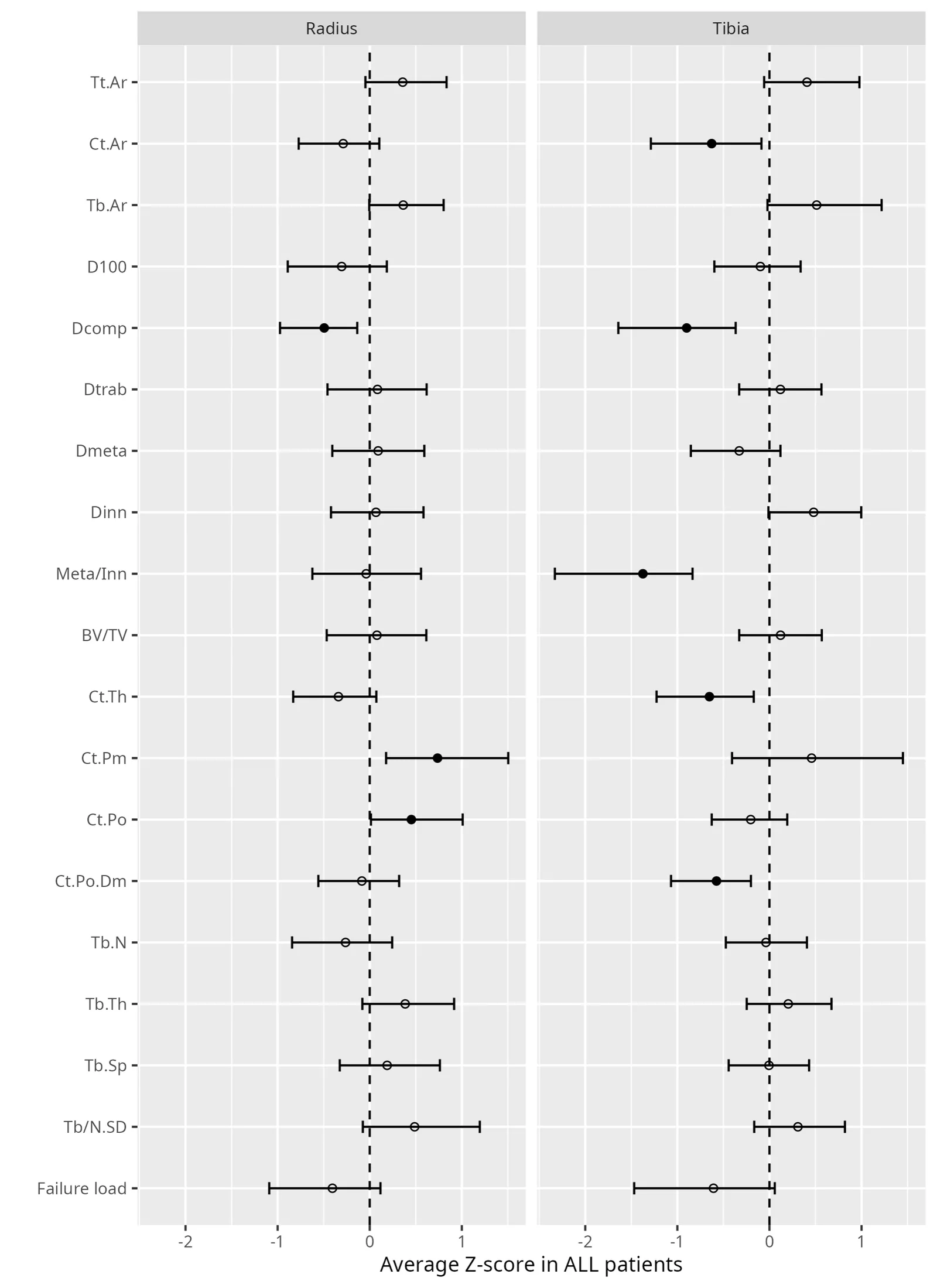
Average z-scores for HR-pQCT parameters at the radius and tibia for pediatric ALL patients. Reference curves were generated based on the 64 healthy controls and a non-parametric bootstrap was used to account for the uncertainty in the estimation of z-scores when quantifying the difference between the two groups. Tt.Ar, total area; Ct.Ar, cortical area; Tb.Ar, trabecular area; D100, volumetric average BMD; Dcomp, cortical BMD; Dtrab, trabecular BMD; Dmeta, meta trabecular BMD; Dinn, inner trabecular BMD; Meta/Inn, ratio of meta to inner trabecular BMD; BV/TV, trabecular bone volume fraction; Ct.Th, cortical thickness; Ct.Pm, cortical perimeter; Ct.Po, cortical porosity; Ct.Po.Dm, cortical pore diameter; Tb.N, trabecular number; Tb.Th, trabecular thickness; Tb.Sp, trabecular separation; Tb/N.SD, trabecular separation standard deviation.

**Figure 2:**
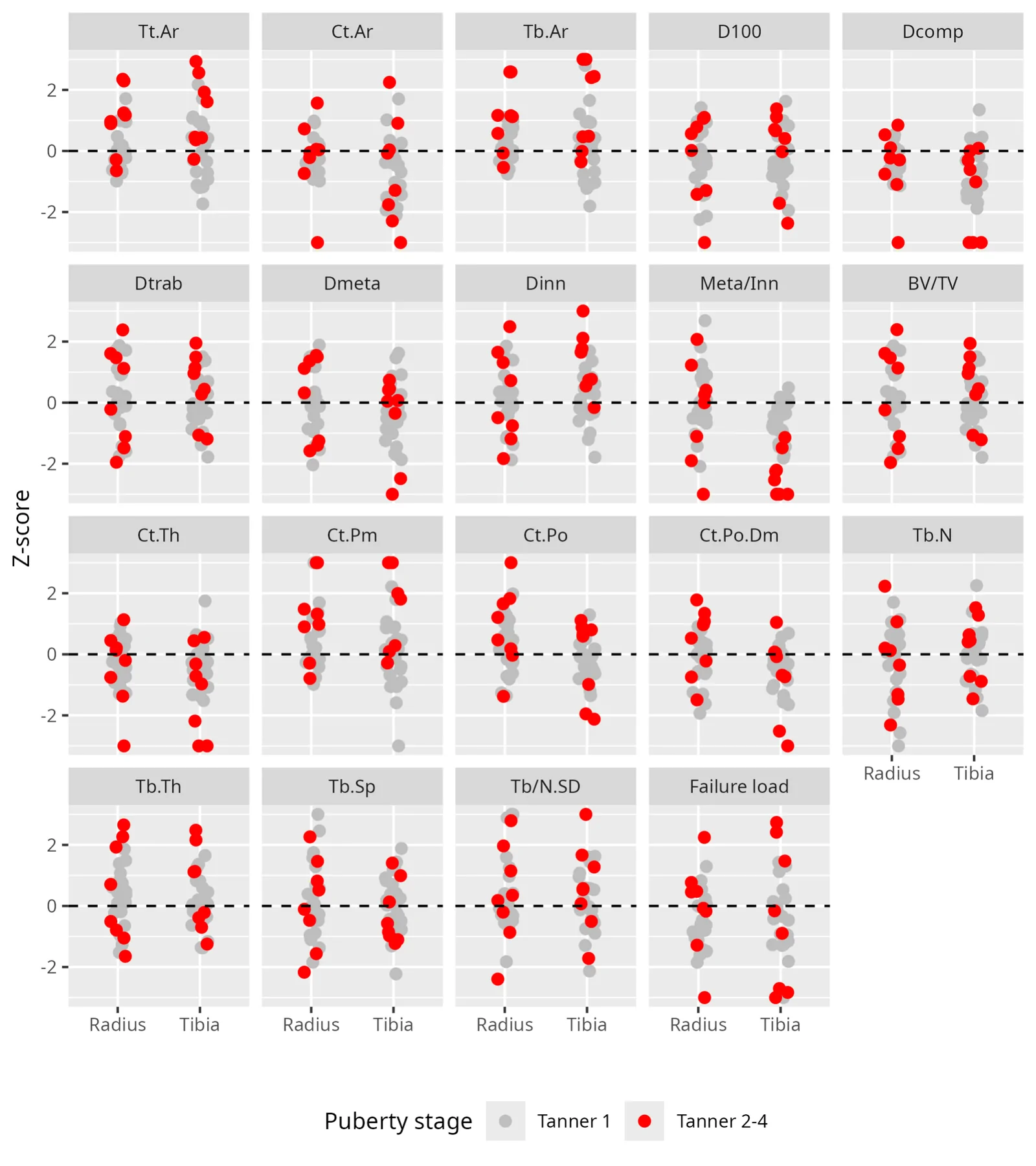
Average z-scores for HR-pQCT parameters in prepubertal and pubertal ALL patients. Reference curves were generated based on the 64 healthy controls and a non-parametric bootstrap was used to account for the uncertainty in the estimation of z-scores when quantifying the difference between the two groups. Tt.Ar, total area; Ct.Ar, cortical area; Tb.Ar, trabecular area; D100, volumetric average BMD; Dcomp, cortical BMD; Dtrab, trabecular BMD; Dmeta, meta trabecular BMD; Dinn, inner trabecular BMD; Meta/Inn, ratio of meta to inner trabecular BMD; BV/TV, trabecular bone volume fraction; Ct.Th, cortical thickness; Ct.Pm, cortical perimeter; Ct.Po, cortical porosity; Ct.Po.Dm, cortical pore diameter; Tb.N, trabecular number; Tb.Th, trabecular thickness; Tb.Sp, trabecular separation; Tb/N.SD, trabecular separation standard deviation.

**Figure 3:**
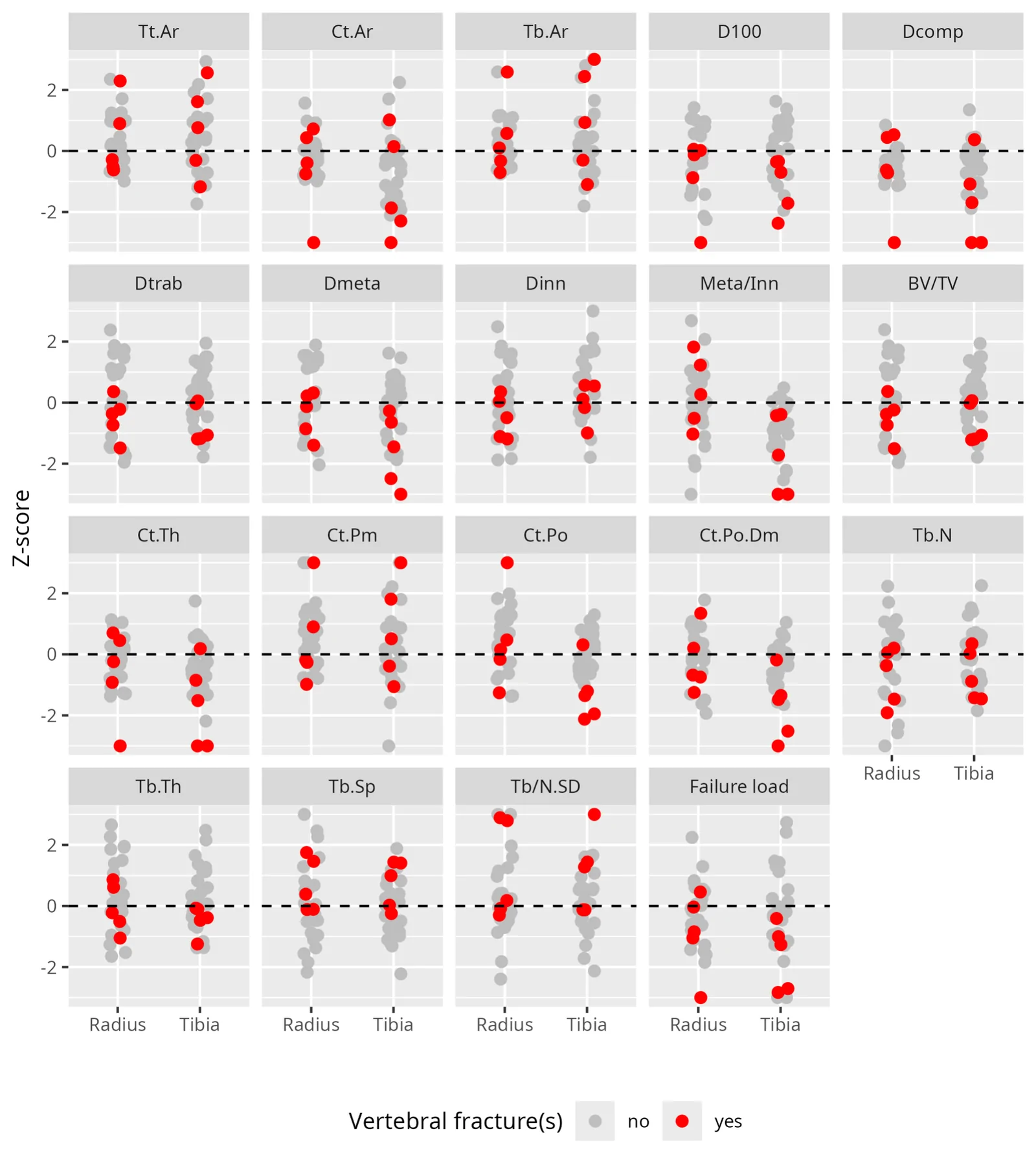
Average z-scores for HR-pQCT parameters in patients with and without vertebral fractures. Reference curves were generated based on the 64 healthy controls and a non-parametric bootstrap was used to account for the uncertainty in the estimation of z-scores when quantifying the difference between the two groups. Tt.Ar, total area; Ct.Ar, cortical area; Tb.Ar, trabecular area; D100, volumetric average BMD; Dcomp, cortical BMD; Dtrab, trabecular BMD; Dmeta, meta trabecular BMD; Dinn, inner trabecular BMD; Meta/Inn, ratio of meta to inner trabecular BMD; BV/TV, trabecular bone volume fraction; Ct.Th, cortical thickness; Ct.Pm, cortical perimeter; Ct.Po, cortical porosity; Ct.Po.Dm, cortical pore diameter; Tb.N, trabecular number; Tb.Th, trabecular thickness; Tb.Sp, trabecular separation; Tb/N.SD, trabecular separation standard deviation.

Average z-scores for HR-pQCT parameters in ALL patients are provided in **Figure 1**. Significant differences in mean HR-pQCT z-scores between ALL survivors and healthy controls (used to build norms) are consistent with the absolute results provided above. Possible trends in average z-scores stratified by sex (a) and age (b) are shown in **Supplementary Figures a, b**. For visual comparison, z-scores of HR-pQCT parameters of pubertal patients are highlighted in **Figure 2**. No obvious trends in z-scores related to puberty were identified visually. Additionally, z-scores for HR-pQCT parameters in patients with VF are highlighted in **Figure 3**. Visually, pediatric ALL patients with VF show predominantly negative z-scores for the ratio of meta to inner trabecular BMD and cortical pore diameter at the tibia and for total BMD, trabecular BMD, trabecular bone volume fraction, trabecular number, and failure load at the radius and tibia compared with patients without VF.

Although LSBMD DXA z-scores were within the normal range, a considerable proportion of pediatric ALL patients exhibited VF and impaired bone health parameters assessed by HR-pQCT. Compared with healthy controls, significant differences were identified mainly in cortical parameters, such as cortical BMD, cortical thickness, and cortical area, predominantly at the tibia but also at the radius.

## 4 Discussion

In adult patients with glucocorticoid-induced osteoporosis from rheumatic disease, similar impairments in HR-pQCT parameters have been identified, including cortical area, cortical BMD, trabecular number, and trabecular separation at the tibia (47). Furthermore, a study in postmenopausal women showed an independent association between multiple and severe VFs and altered cortical bone parameters on HR-pQCT (48). To our knowledge, there are no other studies to date using HR-pQCT to investigate bone health in pediatric patients with a history of ALL. In general, the use of HR-pQCT to assess bone health in children at risk for secondary osteoporosis is still limited (23, 32, 49–52). Possible reasons are limited access to the device, different methodologies of image acquisition, and limited published healthy control data for children (23, 24, 32).

In this cohort of 30 pediatric ALL patients, 5 (17%) had at least one VF, confirming the diagnosis of osteoporosis within 5 years after the end of therapy. These results are comparable to previous studies: Winkel et al. showed a cumulative VF incidence of 17.8% (10), and Cummings et al. showed that 26.4% of pediatric ALL patients had VFs within 4 years after the end of therapy (12). Compared with our results (-0.09), mean LSBMD z-scores have been reported to be somewhat lower in other studies, with z-scores within the lower-normal range (-1.1, -1.2, -0.6 SDS) (10, 12, 14). In the study of Cummings et al., lower baseline LSBMD from DXA was one predictor of VF risk in pediatric ALL patients. Similar trends can be identified in this cohort when LSBMD z-scores are stratified by VF (-0.78 vs. 0.05).

In bone health-related laboratory measures, only 15% of ALL patients had evidence of vitamin D deficiency. This proportion seems relatively low compared with some reports in the general pediatric population and in pediatric ALL survivors (53, 54). Possible explanations may include increased awareness and changes in supplementation practices following survivorship recommendations (30% of ALL patients were taking vitamin D supplements compared with 12.5% of healthy controls). This may influence the diagnostic imaging results and may partially explain the relatively preserved bone health parameters observed with DXA.

With the goal of minimizing radiation exposure, a recent expert article on glucocorticoid-induced osteoporosis in children discussed the option of performing a lateral spine radiograph only in patients with low BMD from DXA and back pain (55). On the other hand, several studies, including ours, show that a normal BMD from DXA does not rule out osteoporosis in pediatric ALL patients and VFs are asymptomatic in almost half of these patients (10, 11, 56). These data suggest that DXA alone may not be sufficient to adequately identify all pediatric ALL patients at risk for bone health complications.

In adults, several HR-pQCT parameters, including total, cortical, and trabecular BMD, cortical thickness, cortical area, cortical porosity, and failure load, are independent predictors of fracture risk (16–18). While statistical significance needs to be confirmed in larger studies, we observed a visual trend toward more negative HR-pQCT z-scores for ALL survivors with VFs compared with those without VFs. This may suggest that other low-radiation imaging techniques, such as HR-pQCT, may help identify children with impaired bone health at risk for secondary osteoporosis even before they experience fractures. Radiation exposure associated with DXA and HR-pQCT is minimal. A combined lumbar spine and total hip DXA examination delivers an effective dose of approximately 6–13 µSv (57), while HR-pQCT imaging of the distal radius and tibia delivers approximately 6–10 µSv (58) using first-generation scanners. Overall, the radiation burden of both imaging modalities is comparable to approximately one day of natural background radiation and is substantially lower than that of a standard chest radiograph.

A key strength of this study is the parallel assessment of different bone health imaging modalities, including lateral spine radiograph (EOS), DXA, and HR-pQCT, in addition to other bone health variables such as nutrition and physical activity. Furthermore, a control group of children and adolescents with a similar age and sex distribution was recruited to provide norms in the absence of published normative data for this age group.

However, this exploratory study has several limitations: although other studies using HR-pQCT in children and adolescents had a similar number of participants (31, 49, 52), the healthy control sample used to generate normative z-scores for our cohort was limited, and the subgroup (n = 5) of patients with VFs was too small for further statistical analysis. Additional studies with larger control samples would be needed to confirm the z-score-derived findings, and a larger patient sample size would be required for further subgroup analysis. The large number of HR-pQCT parameters investigated also raises the problem of inflated type I error due to multiple testing. We report adjusted p-values correcting for multiple testing, which confirmed a decrease in cortical BMD and the ratio of meta to inner trabecular BMD at the tibia of ALL patients compared with healthy controls. However, other exploratory findings did not remain significant following multiple testing correction, and larger studies would be required to investigate differences in other HR-pQCT parameters in more detail. Another limitation is that laboratory measures and additional DXA scans were not performed in the healthy control group for ethical reasons, and published age-specific reference ranges were provided instead.

Nevertheless, our study provides novel insights into structural bone microarchitecture impairment using HR-pQCT in pediatric ALL patients within 5 years after completion of ALL treatment with normal LSBMD z-scores from DXA. These findings suggest that HR-pQCT may be a useful adjunctive tool to better characterize bone health in children at risk for secondary osteoporosis.

## Data Availability

The raw data supporting the conclusions of this article will be made available by the authors, without undue reservation.
