# Supplementary material for: Impaired bone health after pediatric acute lymphoblastic leukemia treatment despite normal DXA: insights from high-resolution peripheral quantitative computed tomography
Source: Front Endocrinol (Lausanne). 2026 Sep 7;17:1850461. doi: 10.3389/fendo.2026.1850461 (PMC13590404; doi:10.3389/fendo.2026.1850461)
Supplement: Supplementary file 1 [file DataSheet1.docx]

**Supplementary Figure:** Average HR-pQCT z-scores stratified by sex (A) and b:y age (B)

**A**

Tt.Ar= total area; Ct.Ar= cortical area; Tb.Ar= trabecular area; D100=volumetric average BMD; Dcomp= cortical BMD; Dtrab=trabecular BMD; Dmeta=meta trabecular BMD, Dinn=inner trabecular BMD; Meta/Inn= ratio meta to inner trabecular BMD; BV/TV= trabecular bone volume fraction; Ct.Th= cortical thickness; Ct.Pm= cortical perimeter; Ct.Po= cortical porosity; Ct.Po.Dm= cortical pore diameter; Tb.N= trabecular number; Tb.Th= trabecular thickness; Tb.Sp=trabecular separation; Tb/N.SD= trabecular separation standard deviation

**B**

Tt.Ar= total area; Ct.Ar= cortical area; Tb.Ar= trabecular area; D100=volumetric average BMD; Dcomp= cortical BMD; Dtrab=trabecular BMD; Dmeta=meta trabecular BMD, Dinn=inner trabecular BMD; Meta/Inn= ratio meta to inner trabecular BMD; BV/TV= trabecular bone volume fraction; Ct.Th= cortical thickness; Ct.Pm= cortical perimeter; Ct.Po= cortical porosity; Ct.Po.Dm= cortical pore diameter; Tb.N= trabecular number; Tb.Th= trabecular thickness; Tb.Sp=trabecular separation; Tb/N.SD= trabecular separation standard deviation
